# Sampling and weighting of the Austrian Psychiatric Prevalence Survey (APPS)

**DOI:** 10.1007/s40211-019-0305-6

**Published:** 2019-04-03

**Authors:** Rainer W. Alexandrowicz, Johann Bacher, Johannes Wancata

**Affiliations:** 1 Institut für Psychologie, Abteilung für Angewandte Psychologie und Methodenforschung, Alpen-Adria-Universität Klagenfurt, Universitätsstraße 65, 9020 Klagenfurt, Austria; 2 Institut für Soziologie, Abteilung für Empirische Sozialforschung, Johannes Kepler Universität Linz, Altenberger Straße 69, 4040 Linz, Austria; 3 Universitätsklinik für Psychiatrie und Psychotherapie, Klinische Abteilung für Sozialpsychiatrie, Medizinische Universität Vienna, Währinger Gürtel 18–20, 1090 Vienna, Austria

**Keywords:** Population representative sample, Stratified sample, Cluster sample, Prevalence of psychiatric disorders, Repräsentative Stichprobe, Geschichtete Stichprobe, Klumpenstichprobe, Prävalenz psychiatrischer Erkrankungen

## Abstract

Mental disorders are common and have severe consequences for the patients, their relatives, and society. Mental health care planning requires precise knowledge of the prevalence of psychiatric disorders and details regarding the provided treatment. Because administrative data lack information on persons not in contact with health services, we need epidemiologic studies delivering nationwide information on the prevalence of psychiatric disorders. This requirement induces the need for adequate sampling procedures to collect reliable data, allowing for accurate estimations of mental health care needs, over- and underprovision. This is the purpose of the Austrian Psychiatric Prevalence Survey (APPS).

The present technical report describes the exact procedure how a nationwide sample has been drawn, adopting a stratified cluster sampling scheme. Because such a complex sampling procedure requires an adequate weighting to obtain unbiased population estimates, this report also contains the exact steps to calculate the corresponding weights. This way, the report not only fosters the full disclosure of the sampling strategy of the APPS, it may also serve as a best practice example for similar endeavours.

**Electronic supplementary material:**

The online version of this article (10.1007/s40211-019-0305-6) contains supplementary material, which is available to authorized users.

## Zusammenfassung

Psychische Erkrankungen stellen nicht nur Betroffene und deren Angehörige vor große Schwierigkeiten und Herausforderungen, sie haben auch maßgebliche gesellschaftliche Konsequenzen. Für eine effektive Planung der Gesundheitsversorgung sind daher exakte Angaben über die Prävalenz verschiedener Erkrankungsbilder sowie bestehende bzw. benötigte Versorgungsmaßnahmen erforderlich. Diese können durch entsprechende epidemiologische Erhebungen bereitgestellt werden. Daraus ergibt sich die Notwendigkeit einer adäquaten Stichprobengestaltung, die nur über komplexe Samplingstrategien realisert werden kann. Für diesen Zweck wurde das „Austrian Psychiatric Prevalence Survey“ (APPS) durchgeführt.

Der vorliegende technische Bericht beschreibt detailliert die Vorgangsweise zur Gewinnung einer österreichweit aussagekräftigen Stichprobe, in der die Prinzipien der geschichteten kombiniert mit einer Klumpenstichprobe angewendet wurden. Ein solches Stichprobendesign erfordert zur Gewinnung unverzerrter Schätzwerte für die abzubildende Population auch die Anwendung einer entsprechenden Gewichtung. Daher werden hier die exakten Schritte zur Bestimmung der erforderlichen Gewichtung dargestellt. Somit soll dieser Bericht nicht nur die technischen Grundlagen des APPS offenlegen, sondern auch als Richtlinie bei der Durchführung ähnlich gelagerter zukünftiger Studien dienen.

## Introduction

For planning adequate mental health care in Austria, the knowledge of the prevalence of psychiatric disorders, the frequency of treatment provided and of the need for treatment is essential [1]. Numerous surveys have shown that mental disorders are common and frequently have severe consequences. For example, increased rates of sickness absence or costs for society due to mental disorders have been reported [2–4]. Based on administrative data some authors have reported increasing rates of unemployment due to mental disorders [5] which resulted in the assumption of an increasing prevalence of mental disorders.

However, administrative data are limited by the fact that they can consider only those who are in contact with health services, but lack information about those not seeking treatment [6]. Thus, the estimation of the frequency of mental disorders among the population and its consequences requires data on the general population. Findings from other countries cannot be transferred, because they differ with respect to their regulatory environment (e.g., health services, training of medical staff or regulations regarding unemployment), population composition, geographical structures, and many other factors.

Therefore, the Austrian Psychiatric Prevalence Survey (APPS) was planned in order to assess the frequency of psychiatric illness, of health service utilization, of the need for psychiatric treatment and the validity of psychiatric screening tools among the general population [7–10].

### The Quest for a “Representative” Sample

Although “representativity” is a frequently used term, we should use it with caution, for it is not underpinned by a clear definition. For example, Stephan [11] tried to narrow down the term, however, arriving rather at a descriptive statement (e.g., “resembles the population”, p. 32) than a mathematically sound definition allowing for deducing concrete action. In this vein, Kish [12] states that “*Representative sampling* is a term easier to avoid because it is disappearing from the technical vocabulary.” (p. 26).

Putting aside the lack of clear definition, we also lack a single universal procedure providing for “representativity” with regard to any population. Rather, the specific structure of the population studied and the research question have to be carefully considered. Kish [12] requires the definition of a population “in terms of (1) content, (2) units, (3) extent, and (4) time.” (p. 7). He exemplifies the terms by means of a consumer survey, in which (1) could refer to all persons, (2) to in family units, (3) the US, and (4) in 1965 (ibid.). For the according specification regarding the present study see Sect. “Target Population and the Sampling Framework”. Fulfilling these requirements cannot be achieved with a convenience sample (the outcome of which is entirely unpredictable), or any other simple sampling procedure. Rather, we have to carefully develop a sampling strategy allowing for an adequate collection of prevalence data.

When seeking meaningful population data in the context of mental health epidemiology, one has to consider carefully, which population characteristics should be represented adequately. The most fundamental variables to be taken care of are a respondent’s sex and age. Next, we have to regard the medical care quality (including also administrative aspects), which we want to cover by distinguishing rural vs. urban population. Although several other aspects would be worth considering as well, we have to limit the requirements to available information (see Sect. “Address Source and Time Frame”).

### Scientific Demand and Standards – the Objective of this Report

To be able to gauge the extent to which study results can be generalized, we have to be aware of how a sample has been drawn. However, information on sampling are frequently incomprehensive or even entirely lacking. For example, Wancata et al. [9] demand a “checklist of methodological requirements […] (e.g. sampling methods, […])” (p. 407). The present report follows this claim and explains in detail the sampling and weighting scheme of the APPS.

The motivation for this report is to give a full account of the intricacy of obtaining a nation-wide representative sample beyond the sparse details usually to be found in articles (claiming “representativity” of their sample not providing convincing evidence, if any). In contrast, the APPS discloses the rationale of how the sample has been drawn in full detail.

This article is structured in the following way: After describing the population to be covered in Sect. “Target Population and the Sampling Framework”, we will explain the sampling procedure in Sect. “The Sampling Procedure”. Because the sampling comprises probability sampling [e.g., 13–15], we have to determine the corresponding weights to take the selection probability correctly into account. This step is described in Sect. “Weighting”.

## Target Population and the Sampling Framework

In terms of Kish’s definition (see Sect. “The Quest for a ‘Representative’ Sample”), the APPS targets (1) the general population aged 18–65 years (2) individually (3) of Austria (4) in 2015–2016. The study design follows the principles of a cross-sectional survey [cf.^16^].

According to the official governmental data base [17], approximately 5.5 million inhabitants of this age group were living in Austria in 2014 (Table 1). Austria is organized in a total of 9 provinces. One of the nine provinces, Vienna (“Wien”), is both a municipality and a province, and at the same time the capital of Austria. Each of the other eight provinces also has a capital. Overall the provinces are organized into a total of 117 political districts (Table 2, column 2) including the capitals, which serve as districts of their own.

It is a peculiarity of the Austrian population distribution that the capital Vienna is by far the largest city in the country, with a population of (approximately) 1.8/8.8 million (21.3%), and 1.2/5.6 million aged 18–65 years (21.3% as well). The second largest city is Graz (the capital of Styria [“Steiermark”]) with a population of 250,000 (i.e., about 1/7 of Vienna) and overall just six cities with a population of 100,000 or more. Therefore, we treat Vienna rather as a province than a municipality, covering 23 districts.

### Sample Size Considerations

Because several analyses involving various procedures are planned, an overall power analysis cannot be performed. Therefore, we calculated as follows: We expect prevalences of the two largest groups *affective disorders* (F3 according to ICD-10 [18]) on the one hand and *anxiety, dissociative, stress-related, somatoform and other nonpsychotic mental disorders* (F4) on the other hand of roughly 10%. Moreover, all analyses shall be performed separately for male and female respondents. Targeting about 50 respondents in these subgroups will result in a total of approximately 1,000. This number matches financial and logistic considerations and it is comparable to similar studies [e.g., 19–21]. To ensure realizing this target and assuming a low response rate, we decided to include a total of 18,000 respondents.

### Address Source and Time Frame

Addresses were bought from one of the largest Austrian address brokers. The data base was a register of Austrian telephone numbers both landline and mobile. Due to factors like change of residence, participation in a mail preference service (“Robinson List”), deceased, etc., we were advised to use the addresses as soon as possible after drawing, otherwise we could face losses. Therefore, we chose to split the 18,000 addresses into three waves of 6000 individual contact records each, which were contacted soon after sampling. The interviews took place from June 2015 until June 2016 with the sampling waves being carried out in June 2015, October 2015, and March 2016.

## The Sampling Procedure

To obtain valid prevalence measures, a sample representative of the Austrian population was required. However, a simple random sample was not feasible, because we did not have access to a population register. Moreover, data acquisition was carried out by trained interviewers; hence, a simple random sample would have likely resulted in prohibitive travelling efforts and costs for the interviewers. Therefore, we decided to apply a cluster sampling scheme based on geographical regions [e.g., 22, Ch. 12]. This scheme allowed for employing regional interviewers and thus kept the travelling expenses within affordable limits.

The patient’s sex is a key-variable determining both the diagnosis of mental illness and the provision of respective health services. We therefore also stratified the sampling with respect to sex (ibid., Ch. 11). Furthermore, because supply differs considerably between urban and rural areas, we also took this information into account, arriving finally at a multi-stage stratified cluster sampling scheme (ibid., Ch. 13).

### Stratification on Province

Due to the federal structure of Austria, the 9 provinces have key responsibilities in certain public health issues. We therefore decided to represent them accordingly in the sample and stratified in a first step with respect to the provinces.

### Cluster Sampling of Districts

Data collection is based on face-to-face interviews, so we have to take the interviewers’ routes to the respondents’ households into account. Cluster sampling requires a full list of predefined clusters from which a random selection can be performed. Our address source disposes of the respondents’ districts; hence, we decided to use this information as primary sampling unit in this step. Austria has a total of 117 districts. Based on logistic and financial considerations, a total of about 40 districts was targeted.

Additionally, the province capitals also play a key role with respect to structural and administrative aspects. Therefore, the following cluster sampling scheme was developed:

- All 8 provincial capitals, being districts of their own, were used. Due to the specific structure of city sizes mentioned in Sect. “Target Population and the Sampling Framework”, this decision was made to represent the urban population accordingly.
- Due to their structural role, the provinces have to be represented evenly. Therefore, the remaining 32 ($=40-8$) districts were selected proportional to the number of districts in each province (see Table 2, column 3).
- After rounding, this calculation resulted in a total of 34 districts to be sampled, 28 rural and 6 urban (see Table 2, columns 4 and 5).
- These 34 districts were sampled at random from the list of all districts per province, excluding the respective provincial capital (except for Vienna, where 6 districts were sampled at random).
- Together with the 8 fixed capital districts, we thus arrived at a total of 42 districts, which are listed in Table 2, last column.

### Stratification According to Province and Sex

The row percentages of Table 1 show that the two sex groups are virtually of equal size if taken across the entire country ($49.95:50.05$), and also the province shares do not exceed a ratio of $51:49$. We, therefore, decided to target the same overall number of men and women.

Next, we wanted to represent the nine Austrian provinces and the two sex groups adequately in the sample. For that purpose, we applied the proportion of men and women within each province (Table 1, columns headed “col %”) to the total sample to be drawn (i.e., 500 men and 500 women, see Sect. “Sample Size Consideration”), obtaining the target sample size for each province. The rounded values are given in the last three columns of Table 1.

**Table 1:** Target population and province/district structure of Austria 18–65 years

	Population	Male		Female		Target Sample		
Province	18–65	$n$	%	$n$	%	Male	Female	Total
Burgenland	186,626	93,754	3.4	92,872	3.3	17	17	34
%	100	50.2		49.8				
Kärnten	356,443	177,772	6.4	178,671	6.4	32	32	64
%	100	49.9		50.1				
Niederösterreich	1,040,527	521,149	18.8	519,378	18.7	94	93	187
%	100	50.1		49.9				
Oberösterreich	924,714	467,415	16.8	457,299	16.4	84	82	166
%	100	50.5		49.5				
Salzburg	348,521	171,982	6.2	176,539	6.4	31	32	63
%	100	49.3		50.7				
Steiermark	792,977	400,308	14.4	392,669	14.1	72	71	143
%	100	50.5		49.5				
Tirol	475,985	237,054	8.5	238,931	8.6	43	43	86
%	100	49.8		50.2				
Vorarlberg	243,353	122,137	4.4	121,216	4.4	22	22	44
%	100	50.2		49.8				
Wien	1,185,085	582,646	21.0	602,439	21.7	105	108	213
%	100	49.2		50.8				
**Total**	**5,554,231**	**2,774,217**	**100.0**	**2,780,014**	**100.0**	**500**	**500**	**1000**
**%**	**100**	**50.0**		**50.0**				

*n* number of inhabitants

Next, we split the province target sample size proportionally to the selected districts according to Table S1 in the supplementary file, columns 4 and 6 (headed “%”). The resulting frequencies for each district are given in the last two columns of Table S1 (rounded to integers). These frequencies were multiplied by 18 (i.e., 6 per wave, see Sect. “Address Source and Time Frame”) to obtain the gross number of addresses to contact.

**Table 2:** Number of districts by province and selection probabilities of districts by province

	Districs in province		Sample				
Province	*n*	%	Rural	Urban	Total	Prob.	Factor
Burgenland	9	7.7	3	$1^{*}$	4	0.44	2.25
Kärnten	10	8.5	3	$1^{*}$	4	0.40	2.50
Niederösterreich	25	21.4	7	$1^{*}$	8	0.32	3.13
Oberösterreich	18	15.4	5	$1^{*}$	6	0.33	3.00
Salzburg	6	5.1	2	$1^{*}$	3	0.50	2.00
Steiermark	13	11.1	4	$1^{*}$	5	0.38	2.60
Tirol	9	7.7	3	$1^{*}$	4	0.44	2.25
Vorarlberg	4	3.4	1	$1^{*}$	2	0.50	2.00
Wien	23	19.7	0	6	6	0.26	3.83
**Total**	**117**	**100.0**	**28**	**14**	**42**	**0.36**	**2.79**

*n* Number of districts in province, *Prob.* Probability

Notes: Asterisks indicate fixed districts (see Sect. “Cluster Sampling of Districts”); The ‘Factor’ column contains the number of districts each selected district stands for; Example: One district from Burgenland stands for 2.25 districts in the entire province. Technically, it is the reciprocal of the selection probability (i.e., in our example $1/0.44=2.25$).

## Weighting

From the procedure described above, we obtained a sample covering a proportional share of respondents for both districts (see Table 2) and respondents (stratified by sex; see Table 3). Regarding districts, the over-all selection probability was 0.36 (however, ranging across provinces from 0.26 to 0.50 because of round-off errors due to the small numbers involved). Regarding respondents, we find a selection probability of 0.018% for both male and female respondents (due to the large numbers involved with remarkably fine-tuned precision).

**Table 3:** Selection probability (in %) of male and female respondents by province

	Probability			Factor		
Province	Male	Female	Total	Male	Female	Total
Burgenland	0.0181%	0.0183%	0.0182%	5,514.9	5,463.1	5,489.000
Kärnten	0.0180%	0.0179%	0.0180%	5,555.4	5,583.5	5,569.422
Niederösterreich	0.0180%	0.0179%	0.0180%	5,544.1	5,584.7	5,564.316
Oberösterreich	0.0180%	0.0179%	0.0180%	5,564.5	5,576.8	5,570.566
Salzburg	0.0180%	0.0181%	0.0181%	5,547.8	5,516.8	5,532.079
Steiermark	0.0180%	0.0181%	0.0180%	5,559.8	5,530.5	5,545.294
Tirol	0.0181%	0.0180%	0.0181%	5,512.9	5,556.5	5,534.709
Vorarlberg	0.0180%	0.0181%	0.0181%	5,551.7	5,509.8	5,530.750
Wien	0.0180%	0.0179%	0.0180%	5,549.0	5,578.1	5,563.779
**Total**	**0.0180%**	**0.0180%**	**0.0180%**	**5,548.4**	**5,560.0**	**5,554.231**

Notes: The probabilities were derived from Table 1; The Factor colums tells us, for how many people of the population each respondent stands (cf. Notes to Table 2).

However, notwithstanding the proportional allocation of districts and sex with respect to province, the sample is not self-weighting, because we performed a random selection of districts based on the number of districts in each province. They were not drawn with a probability proportional to their size, which has to be compensated for. Moreover, all provincial capitals were deliberately included, which can be seen as complete count given the specific city size distribution of Austria (cf. Sect. “Target Population and the Sampling Framework”). Therefore, cities have been selected with a probability of one (with the exception of Vienna, which was treated as a province). Thus, we have to handle the fixed and the randomly selected districts differently.

### Calculating Design Weights

Note: In the following, we will use capital letters to indicate population-based figures and lower case letters for sample-based figures. Stratification is indicated by a superscript in brackets, the subscript $d$ denotes references to the district and subscript $p$ to the province. The symbols $N$ and $n$ denote (true) population and sample frequencies, $M$ projections, and $w$ and $W$ denote weights. The symbols m and f refer to male and female.

We start with the probability of choosing a district at random. This was done with respect to the number of districts of each province. If $K_{p}$ is the number of all districts of a province (col. 2 of Table 2) and $k_{p}$ the number of districts chosen from this province (last col. of Table 2), then the probability of drawing a given district is

1 $$P(\text{district}) = \begin{cases} 1 & \text{district is provincial capital}\\[5mm] \frac{k_{p}-1}{K_{p}-1} & \text{other district except Vienna}\\[5mm] \frac{k_{p}}{K_{p}} & \text{district of Vienna.} \end{cases}$$

Note that for the special case Vienna, there is no provincial capital, hence we used $K$ and $k$ rather than $K-1$ and $k-1$, respectively.

Second, we calculated the probability of a person to be drawn from the selected districts. Due to the stratification according to sex, we had to perform this calculation separately for men and women. If $N_{d}^{(\mathrm{m})}$ is the number of male and $N_{d}^{(\mathrm{f})}$ the number of female inhabitants (aged 18–65) of a district $d$, and $n_{d}^{(\mathrm{m})}$ and $n_{d}^{(\mathrm{f})}$ the respective sample sizes, the according conditional probabilities are

2a $$P(\mathrm{male|district})=\frac{n_{d}^{\mathrm{(m)}}}{N_{d}^{\mathrm{(m)}}}$$

2b $$P(\text{female|district})=\frac{n_{d}^{\mathrm{(f)}}}{N_{d}^{\mathrm{(f)}}}.$$

Hence, the probability of randomly drawing an individual (so far irrespective of the district’s size) is the product

3a $$P(\text{male in district})=P(\text{district})\cdot P(\text{male|district})$$

3b $$P(\text{female in district})=P(\text{district})\cdot P(\text{female|district}).$$

Taking the inverse of Eqs. (3a) and (3b) yields intermediate district projection weights $\widetilde{W}_{d}$,

4a $$\widetilde{W}_{d}^{\mathrm{(m)}}=\frac{1}{P(\text{male in district})}$$

4b $$\widetilde{W}_{d}^{\mathrm{(f)}}=\frac{1}{P(\text{female in district})}.$$

Multiplying the $\widetilde{W}_{d}^{\mathrm{(\cdot)}}$ with the sample size $n_{d}$ of the respective district yields the intermediate district projection $\widetilde{M}_{d}$

5 $$\widetilde{M}_{d}^{\mathrm{(m|f)}}=n_{d}^{\mathrm{(m|f)}}\cdot\widetilde{W}_{d}^{\mathrm{(m|f)}}.$$

(introducing the generic notation (m$|$f) to indicate the separate application of the formula according to the stratification by sex). Eq. (5) lays the foundation to generalize from the chosen districts of a province to the entire province. For that purpose, we have to take the sum of the $\widetilde{M}_{d}$ across all districts of a province $p$ to obtain the (intermediate) province projection $\widetilde{M}_{p}$

6 $$\widetilde{M}_{p}^{\mathrm{(m|f)}}=\sum_{j=1}^{K_{p}}\widetilde{M}_{j}^{\mathrm{(m|f)}}$$

However, these estimates are biased, because we have not yet considered the district size when randomly selecting the districts in Eq. (1). $\widetilde{M}_{p}^{\mathrm{(\cdot)}}$ would over-estimate the respective province totals $N_{p}^{\mathrm{(\cdot)}}$ if we sampled (by chance) rather large districts or under-estimate it if there were more of the small districts of the respective province in our sample (therefore, Eqs. (6) were prefixed “intermediate”).

The district rescaling factor $R_{d}$ corrects for this bias, again taking into account that the provincial capitals (indexed $c$) were deliberately chosen:

7 $$R_{d}^{\mathrm{(m|f)}}=\left\{\begin{array}[]{ll}1&\begin{array}[]{l}\text{district is provincial}\\ \text{capital}\\ \end{array}\\ \displaystyle{\frac{N_{p}^{\text{(m|f)}}-N_{c}^{\text{(m|f)}}}{\widetilde{M}_{p}^{\text{(m|f)}}-N_{c}^{\text{(m|f)}}}}&\begin{array}[]{l}\text{other district excluding}\\ \text{Vienna}\\ \end{array}\\ \displaystyle{\frac{N_{p}^{\text{(m|f)}}}{\widetilde{M}_{p}^{\text{(m|f)}}}}&\text{district of Vienna.}\end{array}\right.$$

We yield the corrected district projections $M_{d}$ by multiplying the intermediate projections (5) by the rescaling factor, i.e.,

8 $$M_{d}^{\mathrm{(m|f)}}=R_{d}^{\mathrm{(m|f)}}\cdot\widetilde{M}_{d}^{\mathrm{(m|f)}}$$

and the province projections $M_{p}$ by taking the sum across all districts of a province, which, as a matter of fact, equal the province size, i.e.:

$$N_{p}^{\mathrm{(m|f)}}=M_{p}^{\mathrm{(m|f)}}=\sum_{j=1}^{K_{p}}M_{d}^{\mathrm{(m|f)}}.$$

To obtain point estimates of population parameters, such as the mean or frequency estimates, for example, we need the respective corrected weights. These are obtainded analoguously by multiplying the intermediate district projection weights by the rescaling factor, i.e.,

9 $$W_{d}^{\mathrm{(m|f)}}=R_{d}^{\mathrm{(m|f)}}\cdot\widetilde{W}_{d}^{\mathrm{(m|f)}}.$$

However, to remain with the sample frequencies, we may simply apply a sample rescaling factor $r$ using the sample size $n$ and the population size $N$,

10 $$r=\frac{n}{N}$$

and obtain the sample district weights

11 $$w_{d}^{\mathrm{(m|f)}}=r\cdot W_{d}^{\mathrm{(m|f)}}.$$

### Target Weighting for Age

Age was not considered in the sampling design, therefore the age distribution of the sample may differ from the respective population distribution. To compensate for effects resulting therefrom, we performed post-stratification weighting using official statistics provided by *Statistik Austria*. We obtained the frequencies of age groups 15–19, 20–24, 25–29, … for both sexes. Although the target population of APPS was 18–65 years, which differs slightly from the limits used in the official statistics available, the practical impact was negligible as it turned out that the observed minimum age in the sample was 20 and only 5 respondents were over 65 (four 66, one 67; these were added to the 60–64 group).

The age weights can be determined directly, because only one target variable is involved [cf.^23^, ch. 7]. For each age group $a$, the age weighting factor $w_{a}$ was obtained separately for male and female respondents using the ratio of the proportion of the sample frequency $n_{a}$ and the respective population frequency $N_{a}$:

12 $$w_{a}^{\mathrm{(m|f)}}=\frac{N_{a}^{\mathrm{(m|f)}}/N}{n_{a}^{\mathrm{(m|f)}}/n}.$$

To consider both sampling and age distribution, the weights (11) and (12) must be multiplied, i.e.,

13 $$w_{da}=w_{d}^{\mathrm{(m|f)}}\cdot w_{a}^{\mathrm{(m|f)}}.$$

Using these weights will exactly reproduce the age distribution of the Austrian population as determined by *Statistik Austria* [17].

## Example Application

Table S2 in the supplementary file provides two examples of the weighting effect. They were compiled with SPSS, using the weight by statement. The examples cover the two demographic variables residents in household and voluntary/unpaid work. Interestingly, we find generally small differences of the weighted compared to the unweighted results. The example.xlsx in the supplement illustrates the application of the weighting formulas for Upper Austria.

Hence, we see that the weights are extremely easy to apply for frequency tables. For more complex analyses and significance tests, one would use the SPSS Complex Samples module, for the standard errors require a modified estimation routine in the context of design weights.

Users of R [24] may choose the survey package [25, 26], for example, which also allows for applying design weights and calculating the correct standard errors and significance tests.

## Discussion

In this report, we presented the sampling rationale and weights calculation for a nationwide epidemiological study in Austria. It comprises a combined strategy involving stratification on province, cluster sampling of districts, stratification on age, and, finally, random sampling.

The procedure has been specifically adapted to the Austrian population structure. It reflects the distributions of inhabitants across the country (organized in provinces and districts) taking into account the specific role of the Austrian provincial capitals. Thus, the chosen procedure provides a sample, which can be considered adequate to obtain results representative for the Austrian population. Moreover, subsequent analyses could focus on indicators for representativity (e.g. by means of a non-responder analysis).

One critical issue is the question, whether the data base used for sampling covers the Austrian population to a sufficient extent. Unfortunately, Austrian law (Meldegesetz 1991, §§ 16a+b) [27] does not allow access to the register of residents (*Zentrales Melderegister*). We, therefore, were left to a commercial vendor. According to a spokesperson, the data base covers approximately 80 % of the Austrian population. The authors of a similar study [28] covering six European countries (not Austria) faced a similar problem in the case of France. They also chose to buy telephone numbers from a commercial vendor and reported a comparable coverage (unliststed rate approximately 16–18%; ibid., p. 9).

If the strategies presented here were to be applied to a country other than Austria, the procedure might simplify, because the complexities of Eqs. (1) and (7) need not be applied. These extra steps were required because of the disproportional distribution of city sizes, which were the motivation to select all province capitals. This extra effort may not be necessary for larger countries or countries with more large cities. Thus, our complex sampling approach might serve as a best-practice example for future studies pursuing a similar target.

## Caption Electronic Supplementary Material

Table S1: Population and target samples of the selected districts aged 18-65 by sex. Notes: Percentages show the city shares per province. Bold entries denote provincial capitals; The numbers in angle brackets denote the official district numbers. Due to rounding errors, the total sample sizes are not 500 and 500 but rather 499 and 504. Table S2: Weighting Examples

Worked example: Applying the weighting formulas to Upper Austria (Oberösterreich)
