# Supplementary material for: Sampling and weighting of the Austrian Psychiatric Prevalence Survey (APPS)
Source: Neuropsychiatr. 2019 Apr 3;33(2):90–7. doi: 10.1007/s40211-019-0305-6 (PMC6561988; doi:10.1007/s40211-019-0305-6)
Supplement: Supplementary file 1 — Table S1: Population and target samples of the selected districts aged 18-65 by sex. Notes: Percentages show the city shares per province. Bold entries denote provincial capitals; The numbers in angle brackets denote the official district numbers. Due to rounding errors, the total sample sizes are not 500 and 500 but rather 499 and 504. Table S2: Weighting Examples [file 40211_2019_305_MOESM1_ESM.pdf]

Electronic Supplementary Material 1
Sampling and Weighting of The Austrian Psychiatric Prevalence Survey (APPS)
(Alexandrowicz, RW, Bacher J, Wancata J) Neuropsychiatrie, **, ***–***

Table S1: Population and target sample sizes of the selected districts aged 18–65 by sex. Notes: Percentages show the city shares per province. Bold entries denote provincial capitals; The numbers in angle brackets denote the official district numbers. Due to rounding errors, the total sample sizes are not 500 and 500 but rather 499 and 504.

		District Population 18–65					Target Sample	
Province	District	m	%	f	%	∑	m	f
B	**Eisenstadt (Stadt)** <101>	4,286	8.2	4,563	8.7	8,849	1	2
B	Neusiedl am See <107>	18,621	35.5	18,468	35.4	37,089	6	6
B	Oberpullendorf <108>	12,223	23.3	11,795	22.6	24,018	4	4
B	Oberwart <109>	17,329	33.0	17,389	33.3	34,718	6	6
	Subtotal Burgenland	52,459	100.0	52,215	100.0	104,674	17	17
K	**Klagenfurt (Stadt)** <201>	30,407	38.8	33,271	41.4	63,678	12	13
K	Hermagor <203>	5,801	7.4	5,599	7.0	11,400	2	2
K	Sankt Veit an der Glan <205>	17,821	22.7	17,312	21.5	35,133	7	7
K	Spittal an der Drau <206>	24,405	31.1	24,256	30.2	48,661	10	10
	Subtotal Kärnten	78,434	100.0	80,438	100.0	158,872	32	32
N	Krems an der Donau (Stadt) <301>	7,863	5.1	7,859	5.0	15,722	5	5
N	**Sankt Pölten (Stadt)** <302>	16,619	10.8	16,923	10.8	33,542	10	10
N	Wiener Neustadt (Stadt) <304>	13,638	8.9	13,981	8.9	27,619	8	8
N	Bruck an der Leitha <307>	13,947	9.1	14,101	9.0	28,048	9	8
N	Gmünd <309>	11,906	7.8	11,247	7.2	23,153	7	7
N	Krems Land <313>	17,913	11.7	17,850	11.4	35,763	11	11
N	Mödling <317>	34,871	22.8	37,277	23.7	72,148	21	22
N	Wien Umgebung <324>	36,415	23.8	37,891	24.1	74,306	22	23
	Subtotal Niederösterreich	153,172	100.0	157,129	100.0	310,301	94	93
O	**Linz (Stadt)** <401>	62,905	34.3	64,701	35.6	127,606	29	29
O	Eferding <405>	10,583	5.8	10,210	5.6	20,793	5	5
O	Linz Land <410>	46,295	25.2	46,170	25.4	92,465	21	21
O	Perg <411>	22,043	12.0	21,129	11.6	43,172	10	10
O	Rohrbach <413>	19,149	10.4	17,494	9.6	36,643	9	8
O	Wels Land <418>	22,666	12.3	22,021	12.1	44,687	10	10
	Subtotal Oberösterreich	183,641	100.0	181,725	100.0	365,366	84	82
S	**Salzburg (Stadt)** <501>	46,210	38.9	49,389	40.1	95,599	12	13
S	Salzburg Umgebung <503>	47,370	39.9	48,074	39.1	95,444	12	13
S	Sankt Johann im Pongau <504>	25,240	21.2	25,617	20.8	50,857	7	7
	Subtotal Salzburg	118,820	100.0	123,080	100.0	241,900	31	32
ST	**Graz (Stadt)** <601>	92,126	48.1	92,575	49.2	184,701	35	35
ST	Hartberg-Fürstenfeld <622>	29,873	15.6	28,550	15.2	58,423	11	11
ST	Murtal <620>	23,257	12.1	22,559	12.0	45,816	9	9
ST	Voitsberg <616>	16,777	8.8	16,472	8.7	33,249	6	6
ST	Weiz <617>	29,454	15.4	28,190	15.0	57,644	11	11
	Subtotal Steiermark	191,487	100.0	188,346	100.0	379,833	72	71
T	**Innsbruck (Stadt)** <701>	41,841	50.7	43,139	52.1	84,980	22	22
T	Landeck <706>	14,615	17.7	14,102	17.0	28,717	8	7
T	Lienz <707>	15,682	19.0	15,258	18.4	30,940	8	8
T	Reutte <708>	10,399	12.6	10,349	12.5	20,748	5	5
	Subtotal Tirol	82,537	100.0	82,848	100.0	165,385	43	43
V	Bludenz <801>	20,115	32.6	19,464	31.9	39,579	7	7
V	**Bregenz** <802>	41,630	67.4	41,480	68.1	83,110	15	15
	Subtotal Vorarlberg	61,745	100.0	60,944	100.0	122,689	22	22
W	Wien 7.Bezirk, Neubau <907>	11,149	9.4	11,752	9.4	22,901	10	10
W	Wien 8.Bezirk, Josefstadt <908>	8,940	7.6	8,863	7.1	17,803	8	8
W	Wien 11.Bezirk, Simmering <911>	30,813	26.0	31,306	25.2	62,119	27	27
W	Wien 17.Bezirk, Hernals <917>	18,653	15.8	18,751	15.1	37,404	17	16
W	Wien 19.Bezirk, Döbling <919>	20,120	17.0	22,357	18.0	42,477	18	20
W	Wien 23.Bezirk, Liesing <923>	28,702	24.2	31,447	25.3	60,149	26	27
	Subtotal Wien	118,377	100.0	124,476	100.0	242,853	105	108
	Total Austria	1.040,672		1.051,201		2.091,873	499	504

Table S2: Weighting Examples

The following two tables show examples of the weighting effect upon the results. The weight

- $W_d^{(m|f)}$ is the district based population projection weight according to Equation (9) of the article,
- $w_d^{(m|f)}$ is the district based sample weight according to Equation (10),
- $w_a^{(m|f)}$ is the target weigth for age according to Equation (12), and
- $w_{da}^{(m|f)}$ is the district based target weight for age according to Equation (13).

**Example 1: Residents in household**	not weighted			weighted by $W_d^{(m|f)}$			weighted by $w_d^{(m|f)}$			weighted by $w_a^{(m|f)}$			weighted by $w_{da}^{(m|f)}$		
Response Category	n	%	valid %	n	%	valid %	n	%	valid %	n	%	valid %	n	%	valid %
solitarily	215	21.3	21.4	1,156,564	20.8	20.9	210	20.8	20.9	205	20.3	20.4	201	20.0	20.0
with spouse or partner	351	34.8	34.9	1,896,757	34.1	34.2	344	34.1	34.2	310	30.8	30.8	303	30.2	30.3
with children and partner	257	25.5	25.5	1,549,527	27.9	28.0	281	27.9	28.0	275	27.3	27.3	300	29.9	30.0
with children but not partner	76	7.5	7.6	387,555	7.0	7.0	70	7.0	7.0	78	7.7	7.7	69	6.9	6.9
with other relatives	68	6.7	6.8	386,515	7.0	7.0	70	7.0	7.0	90	8.9	9.0	92	9.1	9.1
multiperson household (*)	31	3.1	3.1	123,127	2.2	2.2	22	2.2	2.2	39	3.9	3.9	28	2.8	2.8
institutional	1	.1	.1	7,091	.1	.1	1	.1	.1	2	.2	.2	2	.2	.2
other	6	.6	.6	34,143	.6	.6	6	.6	.6	6	.6	.6	6	.6	.6
unknown	1	.1	.1	1,662	.0	.0	0	.0	.0	2	.1	.1	0	.0	.0
Σ valid	1006	99.8	100.0	5,542,941	99.8	100.0	1006	99.8	100.0	1005	99.8	100	1002	99.8	100.0
missing	2	.2		11,290	.2		2	.2		2	.2		2	.2	
Σ	1008	100.0		5,554,231	100.0		1008	100.0		(†) 1007	100.0		1004	100.0	

(*) including flat-mates, but not therapeutic residential community; (†) for one respondent, age was missing and could, therefore, not be considered for age based weighting

**Example 2: Do you practice voluntary/unpaid work on a regular basis?**															
Response Category	n	%	valid %	n	%	valid %	n	%	valid %	n	%	valid %	n	%	valid %
no	717	71.1	74.0	3,877,060	69.8	72.6	704	69.8	72.6	737	73.2	75.7	719	71.6	73.9
yes	252	25.0	26.0	1,462,052	26.3	27.4	265	26.3	27.4	237	23.6	24.3	254	25.3	26.1
Σ valid	969	96.1	100.0	5,339,112	96.1	100.0	969	96.1	100.0	974	96.8	100.0	973	96.9	100.0
missing	39	3.9		215,119	3.9		39	3.9		33	3.2		31	3.1	
Σ	1008	100.0		5,554,231	100.0		1008	100.0		1007	100.0		1004	100.0	
